# Neurodevelopment at seven years and parents' feelings of prematurely born children

**DOI:** 10.3389/fped.2022.1004785

**Published:** 2022-12-05

**Authors:** Clémentine Mercier, Hélène Deforge, Jean-Michel Hascoët

**Affiliations:** ^1^ Division of Neonatology, Maternité Régionale Universitaire, CHRU – NANCY, Nancy, France; ^2^ DevAH 3450, Université de Lorraine, 54500 VANDOEUVRE-LES-NANCY, Vandœuvre-lès-Nancy, France

**Keywords:** neurodevelopmental abnormalities, perinatal network, parental feelings, extreme prematurity, quality of life

## Abstract

**Background:**

The evolution of knowledge and technical advances in neonatal resuscitation have improved the survival of very premature babies. However, the long-term neurodevelopmental prognosis and cognitive and learning abilities are still uncertain.

**Objective:**

This study aimed to evaluate the neurodevelopment and learning abilities of 7-year-old children born prematurely, and their parents' feelings at 8 years of age.

**Patients and methods:**

Data from children born before 33 weeks gestation in a level III maternity hospital and involved in a regional follow-up network were analyzed at 7 years of age. Neurodevelopmental abnormalities were defined as cerebral palsy, hearing or visual impairment, and/or behavioral abnormalities. School performance was evaluated by the EDA test. A parents' questionnaire assessed their feelings about the child's and family's quality of life at 8 years of age.

**Results:**

At 7 years of age, 51% of the 238 children presented neurodevelopmental abnormalities: 3.3% with cerebral palsy, 6.2% with hearing impairments, 50.7% with visual impairments, and 11.3% with behavioral disorders. The children with neurodevelopmental abnormalities had lower gestational age (29.0 ± 2.0 vs. 30.0 ± 2.1 weeks, *p* = 0.003) and more EEG abnormalities during the neonatal period (31.1% vs. 19.8%, *p* = 0.048) than the children without abnormalities. Ninety-four percent of the children with abnormalities were enrolled in normal schools, 33% with special support. In the overall cohort, 31% of the children had all academic performance scores in the normal range of the reference population. At 8 years old, 39% of the parents of children with neurodevelopmental abnormalities felt that their child's situation significantly impacted their quality of life compared to 14% of parents of children without neurodevelopmental abnormality (*p* = 0.022).

**Conclusion:**

Half of children born very prematurely present with long-term neurodevelopmental abnormalities, which their parents feel significantly impacts their quality of life.

## Introduction

Thanks to increased knowledge and technical advances in premature newborn intensive care, the survival of very premature infants has improved. However, their prognosis remains uncertain (1). In 2011, the EPIPAGE 2 national French cohort study showed a significant improvement compared to the 1997 EPIPAGE 1 study, with a 14% increase in the survival rate, without severe morbidity, for preterm babies born between 25 and 29 weeks gestation and 6% increase for babies born between 30 and 31 weeks (2). However, surviving babies without significant initial deficiencies may present with problems in terms of growth, sensory-motor, neuro-psychologic, and cognitive development later in life (3, 4). This untoward evolution may lead to social impacts and affect the family's quality of life with interaction disorders and school difficulties (5, 6).

In this context, a perinatal care network was created for each region in France, making it possible to take care of these vulnerable children early in life and follow them throughout their development up to 8 years of age. As part of the Lorraine regional network program “RAFAEL” (7), we performed a retrospective analysis of data prospectively collected in children born prematurely before 33 weeks gestation between 2010 and 2012. The first objective of this study was to evaluate children's neurocognitive outcome at 7 years of age. Secondary measures of outcome were children's school abilities and an evaluation of the impact of very premature birth on the quality of their family life, the child's behavior, and parents’ feelings at 8 years of age. These evaluations will suggest options for improving the way very premature infants are taken care of for better school achievement and better quality of family life.

## Patients and methods

This is a retrospective analysis of prospectively collected data from a regional cohort study conducted using the medical records of all children born very prematurely before 33 weeks gestation between January 1, 2010, and December 31, 2012. They were cared for in the level III referral center of the Lorraine region and followed in the RAFAEL (Réseau d’Accompagnement des FAmilles En Lorraine) network program.

### Population

Lorraine has approximately 25,000 deliveries annually. All children born prematurely before 34 weeks gestation or with a birth weight <1,800 g may be involved in the program after written agreement from the parents. This program allows them to be followed up to 8 years of age. Follow-up visits and phone interviews enable accurate evaluation of the different stages of development.

At 7 years of age, children had a follow-up visit with a trained registered pediatrician. At 8 years of age, a parents' questionnaire addressed the child's behavior and parents' feelings.

All infants with a follow-up visit at 7 years of age with or without a school-level evaluation with the Evaluation Des fonctions cognitives et des Apprentissages (EDA) (8) and/or whose parents responded to the questionnaire sent at 8 years were included in the analysis. Exclusion criteria were anoxic-ischemic encephalopathy defined as poor neonatal adaptation with APGAR scores <3 and severe early neurological evolution such as seizures, or major neonatal surgery.

### Procedure

The list of children born very prematurely during the study period was obtained from the Medical Information Department of Nancy University Hospital. A table of correspondence (name, date of birth of the newborn, and anonymity number) was established and kept secure in an appropriate place (Office of the Head of Department). Only the children's anonymity numbers appeared on the computer files subsequently created for the study (data collection, processing, and production of results).

All surviving infants were eligible for the regional program at discharge. Infants with data at 7 and/or 8 years of age had their data collected in a standardized manner and kept for analysis. Parameters relevant to the study were defined *a priori* and used the child's medical record from the RAFAEL program.

### Measures

#### Obstetric and neonatal data and parents' education

Obstetric data included multiple births, smoking, alcohol and drug abuse, antenatal corticosteroid use in women, and the occurrence of chorioamnionitis during pregnancy. Neonatal data included gestational age, birth weight, sex, APGAR score, and the occurrence of neonatal disease during hospitalization, such as necrotizing enterocolitis (NEC; above stage IIB of Bell classification) (9) or bronchopulmonary dysplasia [BPD; defined by the RAFAEL network as oxygen therapy or CPAP on day 28 or at 36 weeks post menstrual age (PMA)]. Neonatal neurological impairment was defined by clinical abnormalities (seizures), EEG abnormality, mainly significant dysmaturity background, cerebral MRI carried out at term PMA, and/or brain ultrasound (US): hyperechogenicity lasting more than 7 days, periventricular leukomalacia (10), and intraventricular hemorrhage (IVH) grade I-II or grade III-IV according to Papile classification (11).

Parents' education data were collected for each parent and classified as elementary school, high school, baccalaureate degree, or college.

#### Evaluation at 7 years old: neurodevelopment, academic achievement, and schooling

Neurodevelopmental abnormalities (NAs) were assessed by a trained pediatrician during an in-person visit. NAs were defined as cerebral palsy, hearing or visual impairment, and/or behavioral problems. Hearing and visual disorders were defined as hearing aids or correctives glasses and/or follow-up by a specialist physician or in a specialized institution. Behavioral problems were defined by the pediatrician as suspected or diagnosed autism spectrum disorder, excessive agitation disrupting learning, anxiety, or requiring specific management.

The weight delta Z-score was defined as the difference between the weight Z-score at discharge and weight Z-score at 7 years of age.

A certified neuropsychologist or pediatrician assessed cognitive functions and learning using the EDA (12). Scores in different learning domains (total reading, dictation, and mathematics), non-verbal functions (graphing, planning, visuospatial reasoning, selective visual attention, and constructive praxis), and verbal functions (lexical evocations, lexical and syntax comprehension) were carried out for each child at the 7-year visit. The test was adapted to the child's current school level (first or second grade) and, for the first grade, the trimester was also considered. Children who were unable to achieve the EDA at their corresponding grade level due to various difficulties but passed the inferior level were considered as “grade limited”.

Data on the child's school environment included schooling in an ordinary environment, specialized support network for children in difficulty, presence of an educational team, or education assistant.

#### Parents' feelings

Scores regarding parents' feelings were created for the study using the existing follow-up questionnaire sent to parents at 8 years old.

##### Health score

Seven items were related to parents' views of their child's overall health (excellent, good, average, or weak) and development (normal, early, a little bit late, or very late). Concerns about language, behavior, and coordination were also noted. In addition, difficulties relating to teachers' lack of awareness of prematurity or misunderstanding of the family environment were collected. The health score ranged from 0 to 11. The higher the score, the more difficulties the family had with the child's health. A score of 1 at the most was considered a good health evaluation by the parents.

##### Behavioral score

Sixteen items concerned parents' opinions about their child's behavior in the community. The child could be hyperactive, had difficulty concentrating and maintaining attention, fights, was angry, lonely, unhappy, worried, anxious, harassed by other children or fearful. Conversely, he may be considered friendly and appreciated by other children, empathetic, obedient, and shared easily. For each item, parents could answer often, sometimes, or never. The behavioral score ranged from 0 to 32. The higher the score, the more difficult the behavior in the community. A score below 4 was considered normal behavior by the parents.

##### Quality of life score

Finally, four items dealt with the parents' overall assessment of the child's and family's quality of life: no impact on family life (no points), moderate stress with an impact on family life (1 point), concern and major stress with a significant impact on family life (2 points), or a hefty impact that has disrupted the family (3 points). The maximum quality of life score was 3.

### Statistical analysis

Normally distributed data are presented as mean values with standard deviation (SD), and non-normally distributed data are presented as the median and interquartile range (IQR). A chi-squared test or Fisher exact test was performed when appropriate to compare categorical variables between groups. Continuous variables that were not normally distributed were compared between groups by the Mann-Whitney U test. EDA scores were evaluated based on reference values for French children in the same school grade, allowing calculation of a Z-score for each domain. Two-sided *P*-values <0.05 were considered significant. Statistical analyses were performed in SYSTAT 13 (2009; Systat Software Inc., San Jose, CA, USA).

This study was approved by the Institutional Review Board (Number 2020PI166-82) and is registered at ClinicalTrials.gov (NCT04607109).

## Results

**Figure 1:**
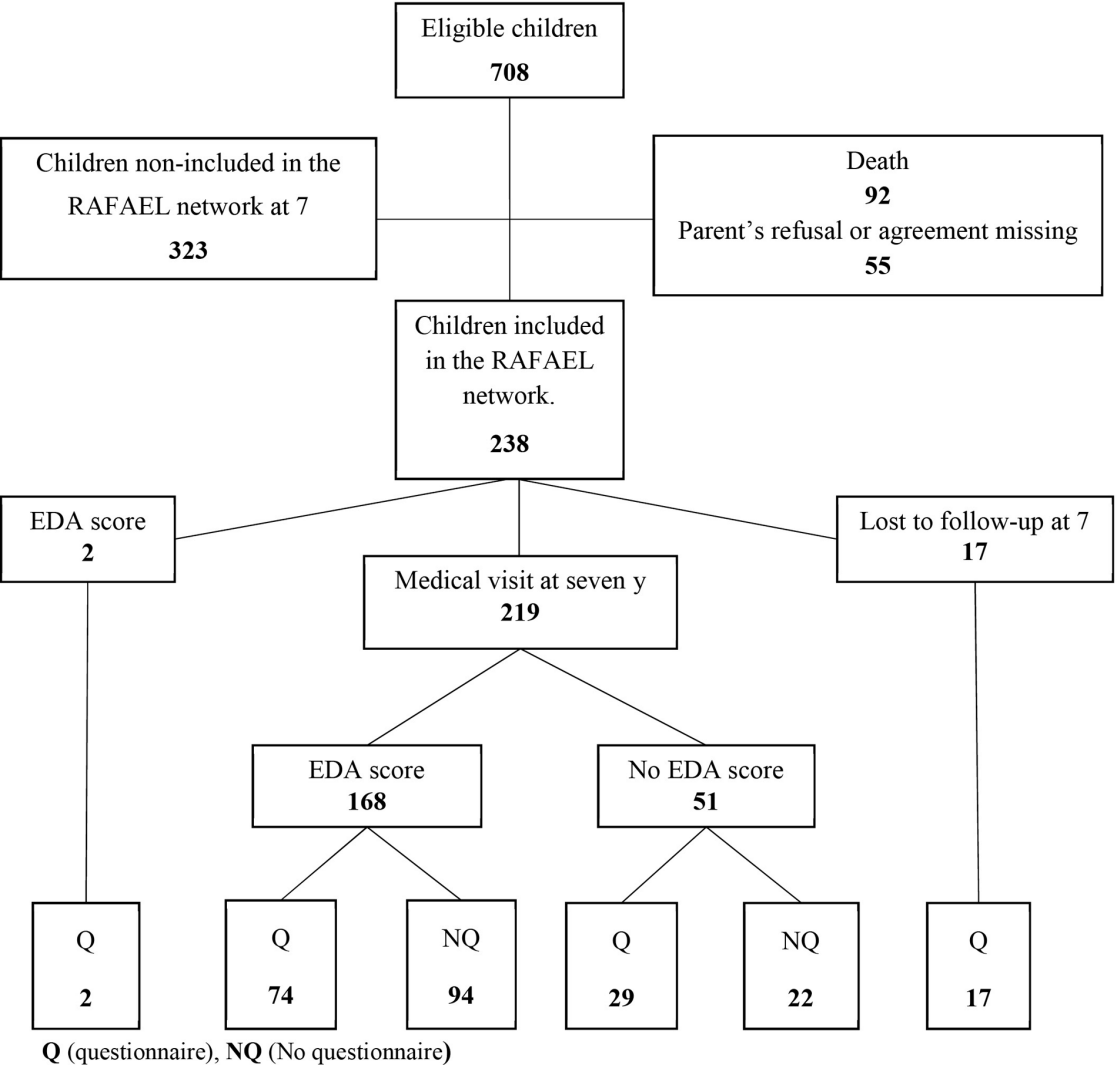
Flowchart of children born before 33 weeks and followed up in the RAFAEL network from 2010 to 2012.

A total of 780 children were born very prematurely in Nancy between 2010 and 2012, and 238 children were included in this study (Figure 1); 323 children were not included because they were not examined at 7 years of age or did not have an EDA evaluation or a parental questionnaire at 8 years of age. The excluded population did not have a significantly different gestational age or hospitalization duration from the studied infants. However, there was a significant difference in birth weight (*p* = 0.031) and excluded infants were more often outborn (*p* = 0.016) than the studied infants (Table 1).

**Table 1:** Comparison of the population included in the study and the population excluded from the study.

Characteristics	Population included (*N* = 238)	Population not included (*N* = 323)	*P*-value
Outborn	13.4%	21.4%	**0**.**016**
Discharged by transfer	58.4%	54.8%	0.395
GA (weeks)	29.5 (2); 30	29.7 (1.9); 30	0.359
Birth weight, g	1,261 (379); 1210	1,324 (375); 1320	**0**.**031**
Duration of hospitalization, days	47 (28); 44	46 (28), 41	0.443

Values are given as the mean (standard deviation) with the median after the semi-colon unless otherwise indicated.

### Neurodevelopmental abnormalities at 7 years of age and perinatal factors

Children's general characteristics at 7 years of age are presented in Table 2A–C. Fifty-one percent of them (*n* = 122) presented with NAs, including 3.3% (*n* = 4) with cerebral palsy, 6.2% (*n* = 8) hearing disorders, 50.7% (*n* = 62) visual disorders, and 11.3% (*n* = 14) diagnosed with behavior disorders. Children with NAs had a lower gestational age (*p* = 0.003), lower birth weight (*p* = 0.03), and longer hospital stays (*p* = 0.001). EEG abnormalities were also more common in children with NAs (*p* = 0.048; Table 2B).

**Table 2:** Preterm infants involved in the rafael network at 7 years of age.

A. Obstetrical data				
Characteristics	Population (*N* = 238)	ND abnormalities (*N* = 122)	No ND abnormalities (*N* = 116)	*P*-value
Multiple birth	73 (30.7%)	35 (28.7%)	38 (32.8%)	0.496
Toxics	62 (27.2%)	34 (29.3%)	28 (25%)	0.465
Tobacco	47	27	20	0.211
Alcohol	3	2	1	
Cannabis	0	0	0	
Drug abuse >2 drugs	8	2	6	
Appropriate antenatal corticosteroids	132 (57.4%)	69 (59.5%)	63 (55.3%)	0.518
Chorioamnionitis	12 (5.2%)	8 (6.6%)	4 (3.4%)	0.301
Values are given as *n* (%).				
**B. Perinatal Data**				
**Characteristics**	**Population (*N* = 238)**	**ND abnormalities (*N* = 122)**	**No ND abnormalities (*N* = 116)**	***P*-value**
GA, weeks	30 (2.1)	29 (2.0)	30 (2.1)	**0.003**
Birth weight, g	1,265 (382)	1,208 (356)	1,326 (400)	**0.031**
Weight at discharge, g	2,653 (467)	2,696 (487)	2,607 (442)	0.640
Male	140 (58.8%)	75 (61.5%)	65 (56%)	0.394
APGAR score				
<4	54 (23%)	27 (22.5%)	27 (23.9%)	0.73
4–6	103 (44%)	56 (46.7%)	49 (41.6%)	
>6	76 (32%)	37 (30.8%)	37 (34.5%)	
NEC	16 (6.8%)	7 (5.7%)	9 (7.8%)	0.522
BPD	40 (16.8%)	22 (18.0%)	18 (15.5%)	0.604
Neonatal neurological impairment	95 (40.1%)	55 (45.5%)	40 (34.5%)	0.085
Seizures	3 (1.3%)	2 (1.6%)	1 (0.9%)	0.586
Brain US	65 (27.3%)	36 (29.5%)	29 (25%)	0.435
Grade 3 or 4 IVH	14	9	5	
Grade 1 or 2 IVH	35	21	14	
Leukomalacia	18	9	9	
Grade 3 or 4 IVH+ Leukomalacia	3	2	1	
Grade 1 or 2 IVH+ Leukomalacia	3	1	2	
EEG abnormalities	60 (25.5%)	37 (31.1%)	23 (19.8%)	**0.048**
MRI abnormalities	43 (53.8%)	29 (59.2%)	14 (45.2%)	0.220
Values are given as mean (standard deviation) or *n* (%).				
**C. Social data**				
**Characteristics**	**Population (*N* = 238)**	**ND abnormalities (*N* = 122)**	**No ND abnormalities (*N* = 116)**	***P*-value**
Fathers’ education level				
Elementary school	4 (2.4%)	2 (2.4%)	2 (2.5%)	0.384
High school	61 (36.3%)	35 (42.2%)	26 (30.6%)	
Bachelor degree	40 (23.8%)	16 (19.3%)	24 (28.2%)	
College	63 (37.5%)	30 (36.1%)	33 (38.8%)	
Mothers’ education level				
Elementary school	4 (2.3%)	2 (2.2%)	2 (2.3%)	0.866
High school	47 (26.9%)	25 (28.1%)	22 (25.6%)	
Bachelor degree	40 (22.9%)	28 (20.2%)	22 (25.6%)	
College	84 (48%)	44 (49.4%)	40 (46.7%)	
At 7 years of age				
Weight, kg	22.9 (4.3)	23 (4.6)	22.8 (4)	0.803
Weight Z-score	−0.08 (1.1)	0.03 (1.2)	0.06 (1)	0.840
Weight delta Z-score	1.3 (1.2)	1.3 (1.3)	1.2 (1)	0.283
EDA test taken	170 (71.4%)	95 (77.9%)	75 (64.7%)	** **
Normal schooling	204 (95.8%)	113 (94.2%)	91 (97.8%)	0.185
Special support at school	52 (24.5%)	39 (32.8%)	13 (14%)	**0** **.** **002**

Values are given as *n* (%) or mean (standard deviation).

Seven children presenting with cerebral palsy at 7 years of age had more frequent abnormalities on EEG (57.1% vs. 42.9%, *p* = 0.058), brain US (71.4% vs. 28.6%, *p* = 0.01), and brain MRI (100% vs. 0.0%, *p* = 0.02). Children diagnosed with behavioral disorders at 7 years old were more often male (83% vs. 16%, *p* = 0.01) and tended to have a longer hospital stay (78 vs. 65 days, *p* = 0.056). No significant differences in neonatal features were found for hearing and visual disorders.

### Neurodevelopmental abnormalities and children's characteristics at 7 years

Ninety-four percent of the children with NAs were in their expected grade in normal schooling, but 33% of them required special support (Table 2C). Fourteen percent of the children with cerebral palsy, 15% with visual impairments, 5% with hearing impairments, and 17% with a behavioral impairment were enrolled in a special school. There was no impact of the parents' education level on the different parameters (Table 2C).

### School results

Seventy-one percent of the children completed the EDA test: 12%, 40%, and 48% were in 1st grade-1st trimester, 1st grade-3rd trimester, and 2nd grade, respectively. Due to difficulties in the various parameters of the EDA, 14.8% of the children were unable to complete the EDA of their corresponding grade level and were grade-limited children.

**Figure 2:**
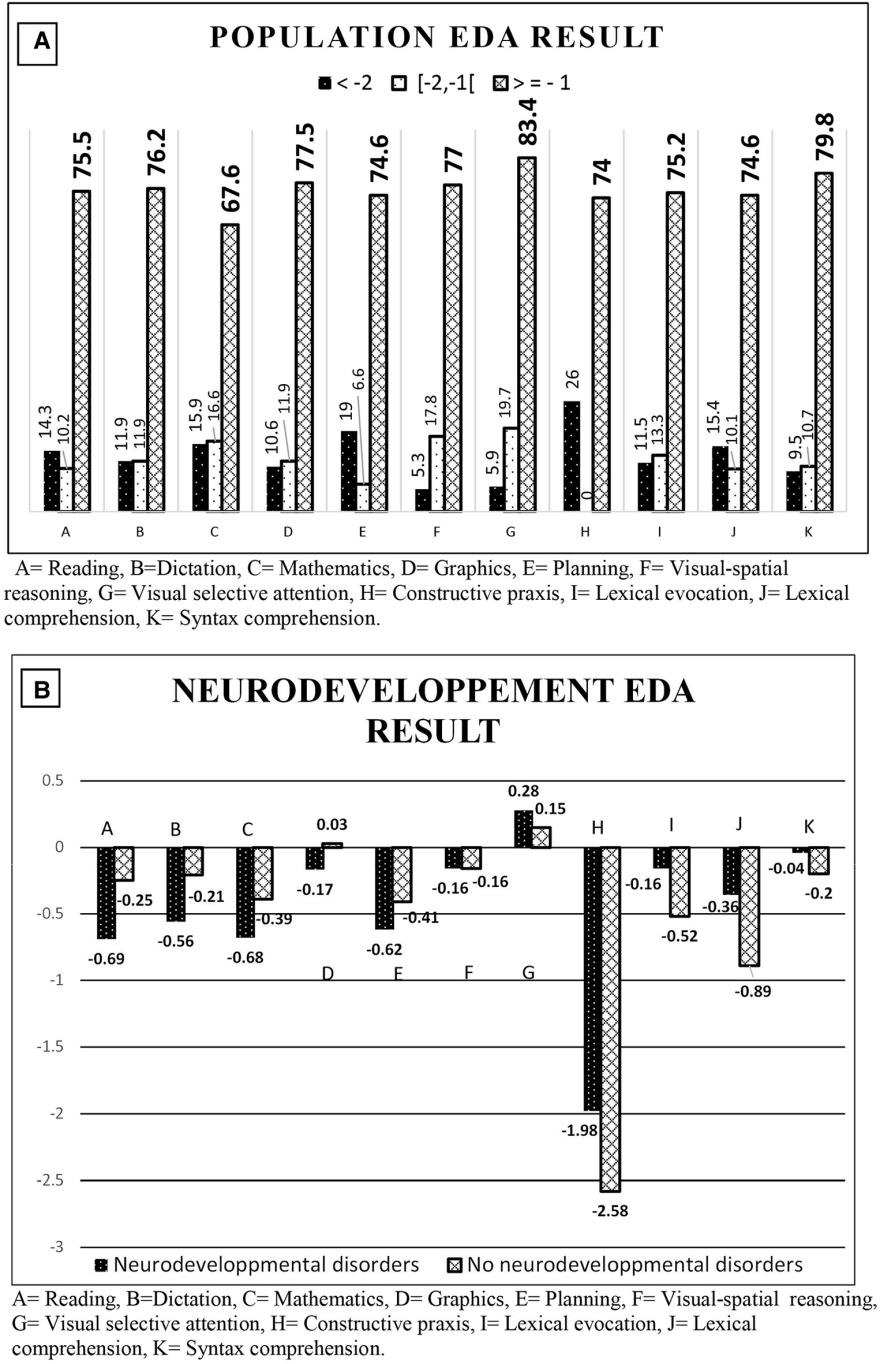
EDA Z-scores. (**A**) Percentage of population based on EDA Z-score. (**B**) Mean Z-scores for the EDA parameters of all grade levels based on neurodevelopmental abnormalities.

Overall, >68% of the children had Z-scores >−1.0 for the 11 parameters individually (Figure 2A). However, the proportion of children with all scores in the normal range was much lower: 28% for the children with NAs and 35% for the children without NAs (*p* = 0.271). Children with NAs tended to have lower Z-scores in reading (*p* = 0.06) and math (*p* = 0.08) than the children without NAs. None of the other parameters were significantly different (Figure 2B). However, the mean Z-scores in reading, visual-spatial reasoning, visual selective attention, constructive praxis, lexical comprehension, and syntax comprehension were significantly associated with the mother's level of education (Table 3).

**Table 3:** Mean (min-max) Z-scores of the EDA according to the mother's education level.

	College	Bachelor degree	High school	Elementary school	*P*-value
Reading	0.32 (−3.52; 1.87)	−0.09 (−9.60; 1.87)	−0.96 (−5.00; 1.23)	−4.77 (−10.0; 1.54)	**0.05**
Dictation	0.27 (−2.06; 2.06)	−0.21 (−5.38; 1.77)	−0.55 (−6.18; 2.06)	−3.07 (−7.29; 1.47)	NS
Mathematics	0.12 (−8.08; 1.80)	−0.35 (−6.42; 1.28)	−0.54 (−5.58; 1.38	−1.98 (−4.46; 1.38)	NS
Graphism	0.32 (−3.11; 2.48)	−0.32 (−3.11; 2)	−0.06 (−3.11; 2.48)	0.20 (−1.53; 0.58)	NS
Planification	−0.15 (−5.6; 1.28)	0.17 (−5.60; 1.28)	−0.45 (−4.28; 1.28)	0.12 (−0.93; 1.28)	NS
Visual-spatial reasoning	0.27 (−1.60; 2.25)	−0.61 (−3.27; 1.48)	−0.56 (−3.93; 1.62)	0,23 (−1.04; 1.62)	0.01
Visual selective attention	0.73 (−3.84; 6.81)	−0.11 (−3.84; 1.39)	− 0.32 (−3.75; 4.58)	0.34 (−1.34; 1.53)	**0.05**
Constructive praxis	0.51 (−9.00; 1.00)	−2.97 (−39.00; 1)	−2.95 (−39.0; 1)	1.00 (1.00; 1.00)	**0.04**
Lexical evocation	0.34 (−12.78; 2.48)	−0.08 (−6.22; 2.43)	−0.87 (−11.39; 2.48)	−0.08 (−1.94; 1.61)	NS
Lexical comprehension	0.22 (−2.90; 2.23)	−0.35 (−4.14; 1.6)	−1,19 (−13.23; 1.77)	0.13 (−1.9; 2.23)	**0.01**
Syntax comprehension	0.60 (−7.36; 1.73)	0.17 (−3.0; 1.73)	−0,54 (−8.36; 1.73)	−2.86 (−9.07; 1.12)	**0.01**

NS, not significant.

Fifteen percent of the grade-limited children were not able to complete all EDA tests of the age corresponding to their grade. They had a lower birth weight (*p* = 0.02) and tended to be twice as small for their gestational age as the children who completed their EDA tests (*p* = 0.08). The duration of their hospital stay was longer (*p* = 0.04) and, thus, the age at discharge was older (*p* = 0.03; Table 4). Twenty-nine percent had an early neonatal infection compared to 10.6% of the other children (*p* = 0.01), and grade-limited children tended to have more bacterial neonatal infections of nosocomial origin (*p* = 0.09). They had more severe ex-utero growth retardation than the other infants, with a Z-score of −1.7 ± 0.8 vs. −1.2 ± 0.9 (*p* = 0.01) at discharge. There was no significant difference in weight z-score at 7 years (−0.28 ± 0.9 for grade-limited children vs. −0 ± 1.1; *p* = 0.24). Grade-limited children had significantly more difficulties in the following areas: visual-selective attention (*p* = 0.01), syntax comprehension (*p* = 0.03), and graphism (*p* = 0.03). In addition, they tended to have more difficulty in lexical comprehension (*p* = 0.06), lexical evocation (*p* = 0.07), visual-spatial reasoning (*p* = 0.08), and planification (*p* = 0.09; Table 4).

**Table 4:** Characteristics of children who did or did not pass the EDA.

	Grade limited (*N* = 25)	EDA passed (*N* = 144)	*P*-value
GA (weeks)	29 (2.2)	29.7 (2)	0.17
Weight birth, g	1,110 (376)	1,295 (363)	**0**.**02**
Weight at discharge, g	2572.8 (413)	2624.8 (449)	0.52
Age at discharge, days	87.3 (3.2)	85.6 (2.6)	**0**.**04**
Length of stay hospital, days	74 (26)	62 (24)	**0**.**04**
Weight at 7, kg	22.1 (3.8)	23.2 (4.4)	0.42
ND abnormalities	14 (56%)	80 (55.6%)	0.97
Cerebral palsy	2	5	0.27
Visual impairment	12	69	0.97
Auditive impairment	0	7	0.27
Behavioral disorders	2	10	0.87
Normal schooling	24 (100%)	134 (97.8%)	0.46
Special support at school	7 (29.2%)	26 (19.1%)	0.26
Z-scores of EDA parameters			
Reading	0.2 (0.9)	−0.5 (2.1)	0.45
Dictation	0.6 (0.9)	−0.4 (1.9)	0.18
Mathematics	−0.9 (2)	−0.6 (2)	0.47
Graphism	−0.6 (1.4)	0 (1.3)	**0**.**03**
Planification	−1.2 (2.2)	−0.4 (1.7)	0.09
Visual-spatial reasoning	−0.6 (1.3)	−0.1 (1.2)	0.08
Visual selective attention	−0.5 (1.5)	0.4 (1.9)	**0**.**01**
Constructive praxis	−6.1 (12.7)	−1.6 (6.6)	0.17
Lexical evocation	−0.9 (3.1)	−0.2 (2.5)	0.07
Lexical comprehension	−1.3 (3.2)	−0.5 (2.3)	0.06
Syntax comprehension	−0.6 (2.2)	−0 (2)	**0**.**03**

GA, gestational age; Values are given as mean (standard deviation) or *n* (%).

### Neurodevelopmental abnormalities and parents' feelings at 8 years of age

The response rate of the parents of children with NAs was 44.3% for the health score, 45% for the behavioral score, and 45% for the quality-of-life score. There was no difference between the parents who responded and the parents who did not for family history, multiple pregnancy rate (28 vs. 33%, *p* = 0.454 respectively) or the duration of hospitalization (62 vs. 68 days, *p* = 0.118, respectively). However, responding parents had higher education level, 48% vs. 25% (*p* = 0.019) of the fathers and 61% vs. 34% (*p* = 0.001) having college degree.

**Figure 3:**
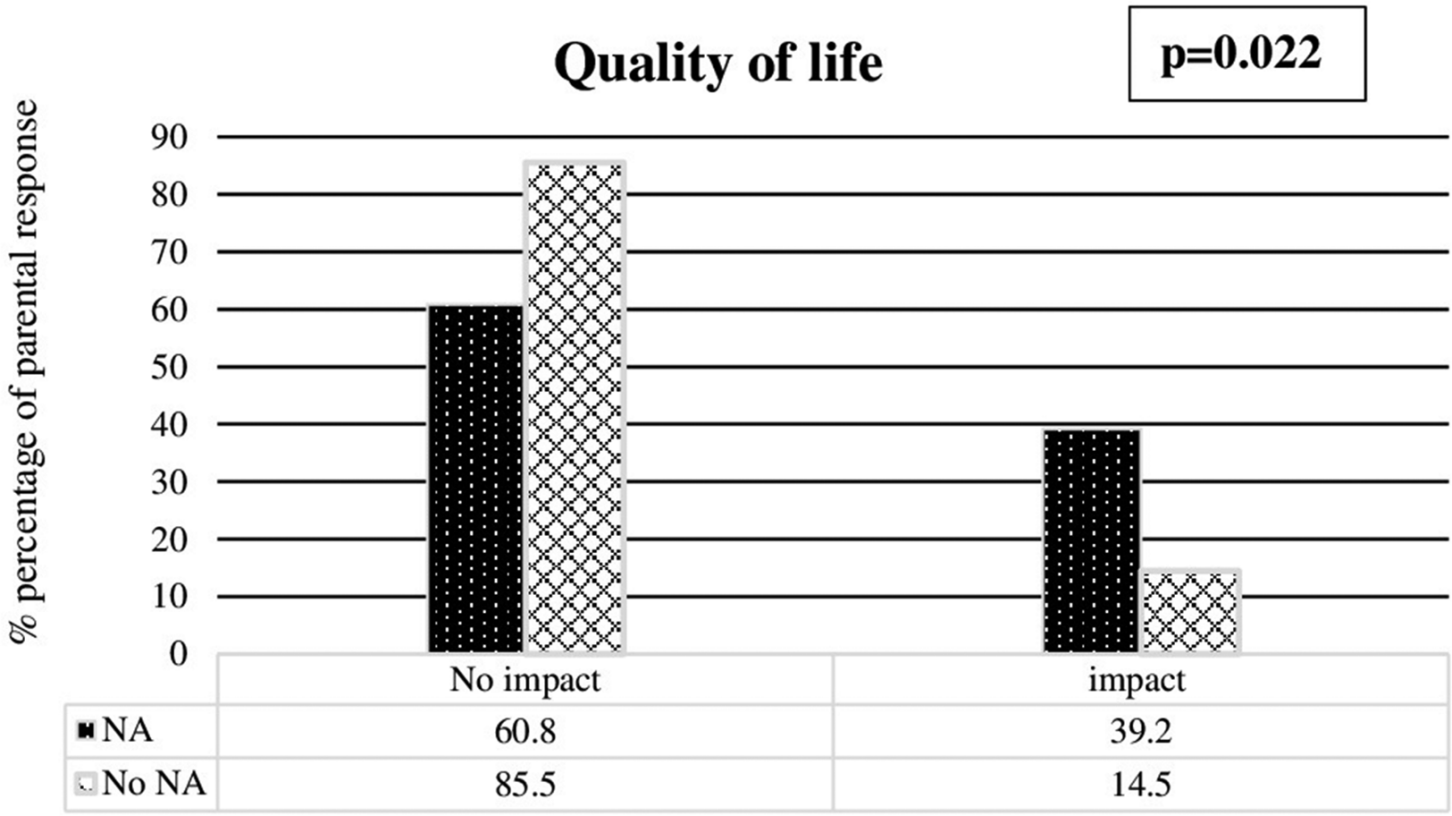
Impact of neurodevelopment at 8 years old on the quality of life scores of the families.

Fifty percent of the parents of infants presenting NAs considered the health score as good vs. 66.2% of the parents of infants without NA (*p* = 0.049). Likewise, 25.9 vs. 42.4% of the parents responded respectively that their child had normal behavior (*p* = 0.046). The impact of NAs was also significantly higher for the parents' quality of life evaluation (Figure 3). Of note, only 33% of the parents of children with hearing impairments had a health score between 0 and 1 compared 60.2% of the parents of children without hearing impairment (*p* = 0.003). No impairment in quality of life was reported by 33% of the parents of children with hearing impairments, compared to 75% for the parents of children without hearing impairment (*p* = 0.03). No significant difference was found in the behavioral score. In addition, no significant difference was found in the health, behavioral, and quality of life scores between the families of children with cerebral palsy, visual, or behavioral disorders and the families of children without disability.

Fifty-four percent of the parents with a health score >1 felt that there was an impact on their quality of life, compared to 46% who reported no impact on quality of life (*p* = 0.001). Of the parents reporting significant concerns and constraints on family life, 87% had a behavioral score >3 vs. 36% with any repercussions on the quality of life (*p* = 0.064).

EDA results did not seem to be associated with health, behavior scores, or the quality of life of the families.

## Discussion

In this study, 51% of very prematurely born children presented with NAs at 7 years of age. The most frequent abnormality was the presence of visual disorders (50.7%). The EPIPAGE 2 French national cohort study was consistent with our results for the rate of cerebral palsy (6.9% vs. 3.3% in our study) and behavioral disorders (7.1% vs. 11.3% in our study), but they reported a rate of moderate or severe vision and hearing disorders of only 1.3% (12). This discrepancy can be explained by the fact that we recorded all abnormalities, including infants with glasses and hearing aids, when the EPIPAGE 2 study only recorded severe sensory disorders leading to a handicap. We decided to record all abnormalities because even corrected problems may have an impact on the quality-of-life of the family. Indeed, 39.2% of the parents of infants with NAs and 14.5% of parents of infants without NAs felt that there was a significant impact on their quality-of-life. Notably, hearing problems were the deficit with the most significant specific impact. No significant difference on the quality of life was found for cerebral palsy, visual impairment, or behavioral abnormalities diagnosed by the pediatrician or by the parents within the group of infants with overall neurodevelopment issues at 7 years of age.

Consistent with the literature, children with NAs had a lower gestational age and lower birth weight (3, 13–15). These children also had significantly more abnormalities on EEG during the neonatal period. EEG recording alone may not show a strong association with long-term NAs. However, a multimodal approach combining term brain MRI, clinical examination, and EEG has been shown to increase the positive likelihood ratio of children at greater risk of NAs at least at 2 years of age (16).

Consistent with the literature, children with cerebral palsy had more abnormalities on brain imaging and were more likely to have pathological EEGs (17, 18).

Children with behavioral problems diagnosed by the pediatrician are more likely to be boys and tend to have a longer stay in the hospital. In contrast to studies showing the influence of parents' education level on NAs (12, 19), we did not find any significant difference with this parameter.

EDA results were encouraging, as only 14.8% of the children were not able to pass the EDA test. Sixty-eight to 83% of the infants scored within the normal range of the reference population for each of the 11 parameters individually. However, only 31% of the children scored within the normal range in all domains, but there was no significant difference for the infants with NAs. This suggests that very prematurely born children do not have homogeneous academic performance. Similarly, mothers' education level influenced specific areas, but not all of them. Children with NAs have been shown to have more difficulty in reading and mathematics (20), which is consistent with our data. The meta-analysis by Allotey et al. (20) included 74 studies comprising 64,061 children and showed that children born prematurely (≤36 weeks) have lower scores in reading [standardized mean differences (SMD): −0.67; 95% CI, −0.87 to −0.47] and math (SMD: −0.78; 95% CI, −1.10 to −0.46). Most children with an NAs at 7 years old were enrolled (94%) in ordinary schools, but more than one-third needed educational support. Specific difficulties need to be identified to optimize the support needed by these children.

In our population, we identified 15% of children as grade-limited, who could not perform the EDA test in their grade due to academic difficulties or behavioral problems but were able to pass the test of the lower grade. The neonatal characteristics of grade-limited children were a lower birth weight, prolonged hospital stay, and more frequent bacterial neonatal infections than other children in the population. They did not have more NAs. They had significantly lower scores in graphism, syntax comprehension, and visual-selective attention. A trend was noted for lexical comprehension and evocation, visual-selective reasoning, and planning. Results from the ELGAN (21) cohort were consistent with our findings. They showed that children with sepsis in the neonatal period performed worse on cognitive assessment, language, academic achievement, and executive function tests without evidence of motor impairment. These children need to be identified early to adjust schooling and psychological support to their needs and improve their outcomes.

Concerning schooling, the children in this study were mostly in normal schools, but only 29% were receiving educational support when 69% had at least one domain below the normal range, showing the complexity of identifying these children. With or without NAs, these children may experience serious difficulties at school without the benefit of support adapted to their needs. Therefore, it seems necessary to strengthen the follow-up of very premature babies by raising awareness of both health and education professionals (22) of the existence of this category of children. In a national survey, 585 teachers and 212 school psychologists completed the PB-KS scale to assess the impact of premature birth knowledge on the learning and development of children born prematurely (23). Teachers had significantly lower knowledge scores than school psychologists (mean 14.7 ± 5.5 vs. 17.1 ± 5.0; *p* < 0.001). Only 16% of teaching staff had received training about preterm birth and more than 90% requested more information. The aim is to limit the negative impact of prematurity on the child and his or her family by anticipating these difficulties and offering appropriate management.

Parents' feelings were studied using three scores created from a self-questionnaire for the study. For parents of children with NAs, all three scores were significantly worse than those of parents of children without NAs. Despite grade-limited children not having more NAs, their parents reported significant impairment in their quality of life compared to the rest of the population. Gire et al. (24). reported that 302 children born <28 weeks gestation who did not have a major disability had quality of life scores significantly lower than those of the reference population.

Our study points out the negative impact of hearing impairment on parental anxiety and family quality-of-life. Hearing impairments lead to impaired language and communication skills and further expose children to relational and behavioral problems. This is consistent with the study by Aras et al., who demonstrated that hearing plays a significant role in a child's development, directly affecting interactions and sociability (23).

Our study has strengths and limitations. First, it is a cohort study evaluating the network performance in a real-life analysis. Few studies have examined parental feelings with regards to long-term neurocognitive assessment. We were able to identify grade-limited children who were free of NAs at 7 years of age but who had lower learning test scores and less school support than other children in the population. Parents of these children were more likely to feel that the status of their infant impacted the quality of life of the family. These infants need to be clearly identified. The limitations of this study are that more than half of the cohort was lost to follow-up. This may be due to the parents' decision or pediatrician availability. Cohort studies are based upon voluntary participation and, therefore, are subject to attrition bias. In our study, the patients lost to follow-up were more often outborn, suggesting fewer health resources for follow-up; they had a higher birth weight but did not have any difference in gestational age or the duration of hospitalization. Therefore, one may speculate that an important difference in outcomes is unlikely. Another limitation is that, within the studied population, only ∼50% of the parents responded to the questionnaire at 8 years. We could not identify specificities for the parents who decided not to answer, but they had predominantly lower education level and the infants had higher neurocognitive difficulties and higher disability rate. As a result, the impact of neurocognitive outcomes on parental feelings may have been underestimated.

Regarding the neonatal records, some data were missing. In addition, the standardized evaluation was not revised since the creation of the network in 2006 and, because some neonatal data are at least 8 years old, they may be outdated. For example, children with pulmonary bronchodysplasia were not satisfactorily identified since the definition of this condition was not updated. Similarly, the EDA battery is a clinical tool for screening verbal, non-verbal, and learning disorders. It is carried out using the paper file provided by the RAFAEL network. However, it highlights the areas in which intervention is desirable. New tools are now available, such as the BMT-i, which is computer-based and similar to the EDA. It will be interesting to improve data collection with computerized files, which would allow better identification of specific problems for each child and prevent the loss of data observed in this study. This would also allow more appropriate and timely responses to individual psychological or educational difficulties, improving the outcomes of the infants, their academic performance, and the quality of life of the families. It would also provide epidemiological data to improve follow-up programs and offer better support to children born very prematurely.

## Conclusion

Children born prematurely require special early and appropriate attention. Although most of them are educated in ordinary environments without any apparent after-effects, a significant proportion still require specific educational support. It is crucial to identify these children and avoid school failure, as it can lead to social failure and impact family interactions and quality of life.

## Data Availability

The original contributions presented in the study are included in the article/Supplementary Material, further inquiries can be directed to the corresponding author/s.
